# Salvianolic acid a attenuates sodium iodate-induced ferroptosis in age-related macular degeneration models via the SLC7A11/GPX4 axis

**DOI:** 10.3389/fphar.2026.1803187

**Published:** 2026-05-25

**Authors:** Jing Wu, Li Luo, Yulu Huang, Jun Li, Xia Li

**Affiliations:** 1 Department of Ophthalmology, Lishui Municipal Central Hospital, The Fifth Affiliated Hospital of Wenzhou Medical University, Lishui, China; 2 Department of Ophthalmology, Chengdu Sixth People’s Hospital, Chengdu, China; 3 The Fifth Affiliated Hospital of Wenzhou Medical University, Lishui, China

**Keywords:** age-related macular degeneration, ferroptosis, retinal pigment epithelium, salvianolic acid A, SLC7A11/GPX4 pathway

## Abstract

**Introduction:**

Oxidative stress-mediated retinal pigment epithelial (RPE) cell injury is a key pathological feature of age-related macular degeneration (AMD). Ferroptosis—an iron-dependent form of regulated cell death driven by lipid peroxidation—has been increasingly identified as a critical executor of RPE degeneration and a key pathogenic mechanism in AMD. This study aimed to investigate the mechanism by which Salvianolic acid A (SalA) alleviates ferroptosis in RPE cells under sodium iodate (NaIO_3_)-induced damage.

**Methods:**

Using *in vitro* (ARPE-19 cells) and *in vivo* (mice) NaIO_3_-induced injury models. We assessed cell viability, ferroptosis markers (iron, lipid peroxidation, glutathione), mitochondrial ultrastructure, and key protein expression via biochemical assays, flow cytometry, transmission electron microscopy, and Western blotting.

**Results:**

SalA pretreatment effectively mitigated NaIO_3_-induced ferroptosis, reducing lipid peroxidation, iron overload, and mitochondrial damage. Mechanistically, SalA upregulated the key ferroptosis regulators SLC7A11 and GPX4. Critically, this protection was significantly attenuated by the SLC7A11 inhibitor erastin, confirming the essential role of this axis. *In mice*, SalA attenuated NaIO_3_-induced retinal structural damage and enhanced SLC7A11/GPX4 expression in retina tissues.

**Conclusion:**

This study identifies SalA as a potent inhibitor of ferroptosis in RPE cells, primarily through activation of the SLC7A11/GPX4 axis. These findings suggest that SalA may hold therapeutic potential for AMD and provide a preliminary pharmacological basis for further exploration in retinal degenerative diseases.

## 1 Introduction

Age-related macular degeneration (AMD) represents a primary cause of irreversible blindness among the elderly population, pathologically characterized by progressive macular degeneration (Fleckenstein et al., 2021; Guymer et al., 2023). Clinically, AMD manifests in two principal subtypes: dry (atrophic) and wet (neovascular). The dry form, which is more prevalent, is pathologically defined by the accumulation of drusen deposits beneath the retinal pigment epithelium (RPE), a process that can advance to geographic atrophy and consequent photoreceptor degeneration (Jager et al., 2008; Plaza et al., 2020). The etiology of AMD is multifactorial, involving a complex interplay of genetic predispositions, chronic low-grade inflammation, and metabolic dysfunction (Datta et al., 2017; Hanus et al., 2015; Stock et al., 2017). Well-established genetic risk factors, such as polymorphisms in the complement factor H (*CFH*) gene, point to a central role of complement pathway dysregulation in disease susceptibility and progression (Klein et al., 2005; Hageman et al., 2005). This, coupled with age-related oxidative stress (which itself can trigger complement activation (Hollyfield et al., 2008)), creates a hostile microenvironment that drives RPE dysfunction, drusen formation, and ultimately, photoreceptor degeneration (Ande et al., 2010).

Beyond these established pathways, accumulating evidence implicates ferroptosis—an iron-dependent form of regulated cell death driven by lipid peroxidation—as a pivotal mechanism in AMD pathogenesis. This link is supported by multiple lines of evidence: elevated iron levels are consistently observed in the retinas of AMD patients (Biesemeier et al., 2015); transcriptomic analyses reveal dysregulation of ferroptosis-related genes in AMD-affected tissues (Zhu and Yu, 2024); and critically, genetic ablation of the key ferroptosis suppressor GPX4 in RPE cells recapitulates features of geographic atrophy (Azuma et al., 2024; Yang et al., 2022). These observations position ferroptosis not merely as a correlative process but as a potential causal effector in RPE degeneration, making its pharmacological modulation a compelling therapeutic strategy, as evidenced by the protective effects of ferroptosis inhibitors in various AMD models (Liu et al., 2024; Xiang et al., 2024). Nevertheless, the precise molecular regulators of ferroptosis in RPE cells and effective pharmacological interventions specifically targeting this pathway remain incompletely understood.

This knowledge gap, coupled with the multi-target and neuroprotective properties of natural compounds, makes them particularly attractive candidates for further investigation (Zheng et al., 2024).

Among various natural compounds with therapeutic potential, Salvianolic acid A (SalA) has emerged as a particularly promising agent. This prominent water-soluble constituent of *Salvia miltiorrhiza* Bunge, demonstrates a broad spectrum of pharmacological activities, including potent antioxidant, anti-inflammatory, and neuroprotective effects (Qin et al., 2019). Previous studies have demonstrated that SalA suppresses NLRP3 inflammasome activation by enhancing Schwann cell autophagy, thereby mitigating inflammatory responses in models of peripheral nerve injury (Fu et al., 2025). It also provides neuroprotection through anti-apoptotic mechanisms and by modulating the PKA/CREB/c-Fos signaling pathway (Yang et al., 2024). Particularly relevant to retinal pathology, SalA has also been shown to directly protect RPE cells against oxidative stress (Mao et al., 2017; Zhang et al., 2014). Despite these advances, its potential role in modulating ferroptosis in RPE cells remains unexplored.

To address this knowledge gap, this study investigates the protective effects of SalA against sodium iodate (NaIO_3_)-induced ferroptosis in RPE cells and elucidates the underlying molecular mechanisms via the SLC7A11/GPX4 axis (Upadhyay and Bonilha, 2024). Using an integrated approach combining *in vitro* and *in vivo* models, molecular biology, and transcriptomic analysis, we demonstrate that SalA attenuates ferroptosis by upregulating SLC7A11 and GPX4 expression, thereby offering a novel therapeutic strategy for AMD.

## 2 Materials and methods

### 2.1 Animal handling and experimental procedures

Eight-week-old male C57BL/6 mice were housed in a controlled environment (22 °C ± 2 °C, 55% ± 10% humidity, 12-h light/dark cycle) with *ad libitum* access to food and water. All experimental protocols were reviewed and approved by the Institutional Animal Care and Use Committee of DR. Can Biotechnology (Zhejiang) Co., Ltd. (Approval No. DRK-2025053035) and were conducted in accordance with the ARVO Statement for the Use of Animals in Ophthalmic and Vision Research. The mice were randomly divided into three groups (n = 6) (Fleckenstein et al., 2021): Control (saline vehicle) (Guymer et al., 2023), NaIO_3_ model, and (Jager et al., 2008) SalA treatment. A solution of SalA was prepared in sterile saline at a concentration of 2.5 mg/mL. It was administered intraperitoneally (i.p.) at a daily dose of 15 mg/kg for 1 week. Mice in the control and NaIO_3_ model groups received equivalent volumes of saline. On day 7, mice in the NaIO_3_ and SalA groups received a single tail vein injection of NaIO_3_ (20 mg/kg), while control mice received saline. SalA treatment was continued for 7 days after NaIO_3_ injection. All mice were euthanized by cervical dislocation under deep anesthesia, and ocular tissues were collected for analysis.

### 2.2 Histological processing and immunofluorescence staining

We fixed enucleated eyes in Davidson’s fixative (Scientific Phygenze, #PH0975) for 24 h at room temperature, then processed them for paraffin embedding and sectioning (5 μm). For histological analysis, we stained deparaffinized and rehydrated sections with H&E. For IF analysis, we performed antigen retrieval on deparaffinized sections, blocked them with 5% BSA/0.5% Triton X-100 (1 h), and incubated them with primary antibodies (GPX4 or SLC7A11, 1:200, 4 °C, overnight). After washing, we applied fluorescent secondary antibodies (Alexa Fluor 488/594, 1:500, 37 °C, 1 h) and counterstained nuclei with DAPI. We captured images using a Leica fluorescence microscope. We also harvested major organs (heart, liver, spleen, lung, kidney) for H&E staining.

### 2.3 Cell culture and treatment

We cultured the human retinal pigment epithelial cell line ARPE-19 (GNHu45, National Collection of Authenticated Cell Cultures) in DMEM/F12 medium containing 10% fetal bovine serum (FBS; Gibco) and 1% penicillin-streptomycin (Invitrogen). Cells were kept at 37 °C in a humidified atmosphere of 5% CO_2_. All experiments utilized cells between passages 3 and 8. Prior to treatment, we seeded cells at appropriate densities and allowed them to reach 70%–80% confluence. To induce an *in vitro* retinal injury model, we exposed cells to a concentration gradient of NaIO_3_ (0.15625–10 mM) for 24 h. For the protection assays, we pretreated cells with SalA (0–4 μM) for 2 h before adding NaIO_3_.

### 2.4 Cell viability assay

We assessed cell viability using the Cell Counting Kit-8 (CCK-8; Sigma) according to the manufacturer’s protocol. Briefly, we plated ARPE-19 cells in 96-well plates at 2 × 10^4^ cells per well. Following treatments, we added 10 μL of CCK-8 solution to each well and incubated the plates for 1 h at 37 °C. We then measured the absorbance at 450 nm on a Bio-Rad microplate reader and analyzed the data using GraphPad Prism 9.0.

### 2.5 Measurement of intracellular ROS

We detected intracellular reactive oxygen species (ROS) levels with 2′,7′-dichlorodihydrofluorescein diacetate (DCFH-DA; Beyotime). After the indicated treatments, cells were loaded with 10 μM DCFH-DA and incubated for 30 min at 37 °C. Following PBS washes, cells were analyzed immediately by flow cytometry (BD Biosciences) with excitation and emission wavelengths set at 488 nm and 525 nm, respectively.

### 2.6 Assessment of oxidative stress markers

We quantified intracellular glutathione (GSH) levels following the protocol of a commercial colorimetric assay kit (Sigma, CS0260). Malondialdehyde (MDA) content, a marker of lipid peroxidation, was determined with the Lipid Peroxidation MDA Assay Kit (Sigma, MAK568). Absorbance readings for both assays were obtained using a microplate reader at the recommended wavelengths.

### 2.7 Lipid peroxidation imaging

We visualized lipid peroxidation using the fluorescent probe BODIPY™ 581/591 C^11^ (Beyotime, SOO43S). Post-treatment, cells were loaded with 5 μM of the probe for 30 min at 37 °C. Subsequently, after PBS washes, they were immediately subjected to imaging on a Zeiss LSM 880 confocal laser scanning microscope.

### 2.8 Cellular iron content measurement

We determined intracellular Fe^2+^ levels with a commercial Iron Assay Kit (Sigma, 1.10004) as per the manufacturer’s guidelines. In brief, cell lysates were combined with iron-releasing reagent and incubated at 60 °C for 2 h. Subsequently, iron detection reagent was added, and after a 30-min incubation, absorbance was read at 550 nm. Final iron concentrations were derived from a standard curve.

### 2.9 Transmission electron microscopy

For ultrastructural examination, we fixed treated ARPE-19 cells in 2.5% glutaraldehyde/0.1 M phosphate buffer (pH 7.4; 2 h, 4 °C). After post-fixation in 1% osmium tetroxide, samples were dehydrated in an ethanol series and embedded in epoxy resin. Subsequently, we generated ultrathin sections (70 nm), stained them with uranyl acetate and lead citrate, and visualized on a ZEISS GeminiSEM 300 TEM (80 kV).

### 2.10 Western blot analysis

We lysed cells using RIPA buffer (Beyotime, P0013B) containing protease inhibitors. After quantifying protein concentration with a BCA assay kit (Beyotime), we separated equal amounts (20–30 µg) by SDS-PAGE and transferred them onto PVDF membranes. The membranes were blocked with 10% non-fat milk in TBST for 1 h at room temperature and then incubated overnight at 4 °C with primary antibodies: anti-GPX4 (ABclonal, A25009; 1:2000), anti-SLC7A11 (Proteintech, 26864-1-AP; 1:2000), anti-FTH1 (Proteintech, 11682-1-AP; 1:2000), anti-ACSL4 (MyBioSource, MBS839159; 1:2000), Anti-TFR2 (Proteintech, 32381-1-AP, 1:2000) and anti-β-actin (Proteintech, 20536-1-AP; 1:5,000). After washing, we incubated the membranes with HRP-conjugated secondary antibodies for 1 h at room temperature. Subsequently, bands were visualized via an ECL detection system (Biosharp) and analyzed for intensity with ImageJ software.

### 2.11 RNA sequencing and bioinformatics analysis

We isolated total RNA from ARPE-19 cells with TRIzol reagent (Invitrogen) and evaluated its quality using an Agilent 2100 Bioanalyzer. We prepared cDNA libraries for sequencing on the DNBSEQ™ platform (MGI-Tech). The raw reads were processed for quality control with FastQC, and adapters were removed with Trimmomatic. We aligned the high-quality reads to the GRCh38 genome using HISAT2 (v2.2.1) and quantified gene expression as FPKM. Using DESeq2, we identified differentially expressed genes (|log_2_FC| ≥ 1, adjusted p < 0.05). Functional enrichment for Gene Ontology was carried out with clusterProfiler, and we created visualizations using ggplot2 in R (v4.2.1).

### 2.12 Statistical analysis

We expressed all data as mean ± standard deviation (SD) from a minimum of three independent replicates. We performed all statistical analyses with GraphPad Prism 9.0 software. We applied Student’s t-test for comparisons between two groups. For analyses involving multiple groups, we conducted one-way ANOVA followed by Tukey’s *post hoc* test. A p-value below 0.05 was considered statistically significant.

## 3 Results

### 3.1 Effect of SalA on viability of ARPE-19 cells

**FIGURE 1:**
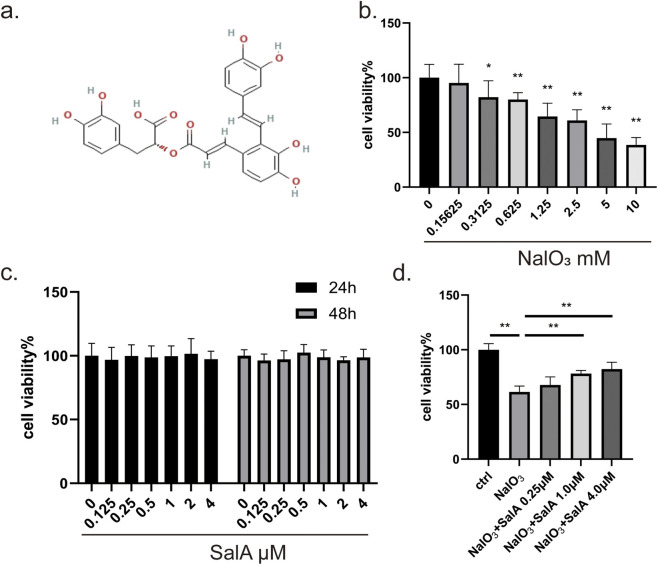
Salvianolic acid A (SalA) protects ARPE-19 cells against sodium iodate (NaIO_3_)-induced injury. **(a)** Chemical structure of SalA. **(b)** The dose-dependent cytotoxic effect of NaIO_3_ (0–10 mM, 24 h) on ARPE-19 cells, determined by CCK-8 assay. **(c)** Assessment of the cytotoxicity of SalA alone at indicated concentrations (0–4 μM) for 24 or 48 h. **(d)** Cell viability following a 2-h pretreatment with SalA (0, 0.25, 1, 4 μM) prior to a 24-h challenge with 2.5 mM NaIO_3_. Data are mean ± SD (n = 3). *p < 0.05, **p < 0.01.

SalA is a polyphenolic compound, whose structure is depicted in Figure 1a. CCK-8 assays showed that NaIO_3_ treatment (0–10 mM, 24 h) reduced ARPE-19 cell viability in a dose-dependent manner, with 2.5 mM inducing approximately 50% cell death (Figure 1b). SalA alone (0–4 μM, 24/48 h) exhibited no significant cytotoxicity (Figure 1c). Notably, a 2-h pretreatment with SalA (0.25–4 μM) significantly attenuated the NaIO_3_-induced decrease in viability, with optimal protection observed at 1 and 4 μM (Figure 1d). Consequently, 4 μM SalA was selected for subsequent experiments.

### 3.2 SalA attenuates NaIO_3_-induced ferroptosis by regulating iron homeostasis

**FIGURE 2:**
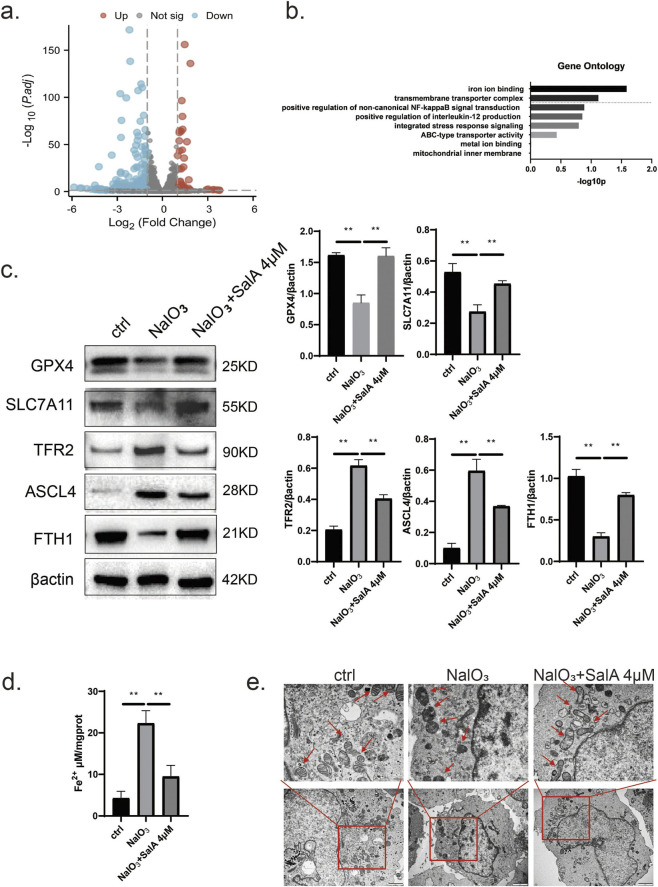
SalA inhibits NaIO_3_-induced ferroptosis by Regulating Iron Homeostasis in ARPE-19 cells. **(a)** A heatmap depicts the gene expression changes (|log_2_FC| ≥ 1, adjusted p < 0.05) induced by NaIO_3_/SalA, with up- and downregulated genes colored red and blue, respectively. **(b)** Enrichment analysis of Gene Ontology terms identified key biological processes, including iron ion binding and the regulation of ferroptosis. **(c)** Western blot analysis of key ferroptosis regulators. NaIO_3_ treatment downregulated the ferroptosis suppressors GPX4, SLC7A11, and FTH1 while upregulating the promoters ACSL4 and TFR2; all these alterations were markedly reversed by SalA co-treatment. **(d)** Quantification of intracellular Fe^2+^ levels using a colorimetric assay. SalA pretreatment significantly attenuated NaIO_3_-induced iron accumulation. **(e)** Transmission electron microscopy revealed the ultrastructural changes in mitochondria. The NaIO_3_-triggered ferroptotic morphology (mitochondrial shrinkage and denser membranes, indicated by red arrows) was ameliorated by SalA. Data are shown as mean ± SD (n = 3 independent experiments). *p < 0.05, **p < 0.01.

To explore the protective mechanism of SalA, we performed RNA sequencing. This analysis identified a total of 326 differentially expressed genes (DEGs; |log_2_FC| ≥ 1, adjusted *p* < 0.05), including 72 upregulated and 254 downregulated genes in SalA-pretreated cells compared to the NaIO_3_ group (Figure 2a). Following this clue from GO enrichment analysis, which highlighted iron ion binding (Figure 2b), we focused on ferroptosis. This was confirmed by Western blot, showing NaIO_3_-induced downregulation of ferroptosis suppressors (GPX4, SLC7A11, FTH1) and concurrent upregulation of promoting factors (ACSL4 and TFR2); these alterations were significantly reversed by SalA co-treatment (Figure 2c). Consistent with these findings, intracellular Fe^2+^ levels were substantially elevated following NaIO_3_ exposure, and this accumulation was inhibited by SalA (Figure 2d). Transmission electron microscopy (TEM) further confirmed ferroptotic ultrastructural changes in the NaIO_3_ group, including mitochondrial shrinkage and increased membrane density (red arrows, Figure 2e), which were markedly alleviated by SalA. Collectively, these data indicate that SalA mitigates NaIO_3_-induced ferroptosis by modulating iron homeostasis and restoring expression of core ferroptosis regulators.

### 3.3 SalA alleviates NaIO_3_-induced oxidative stress and lipid peroxidation

**FIGURE 3:**
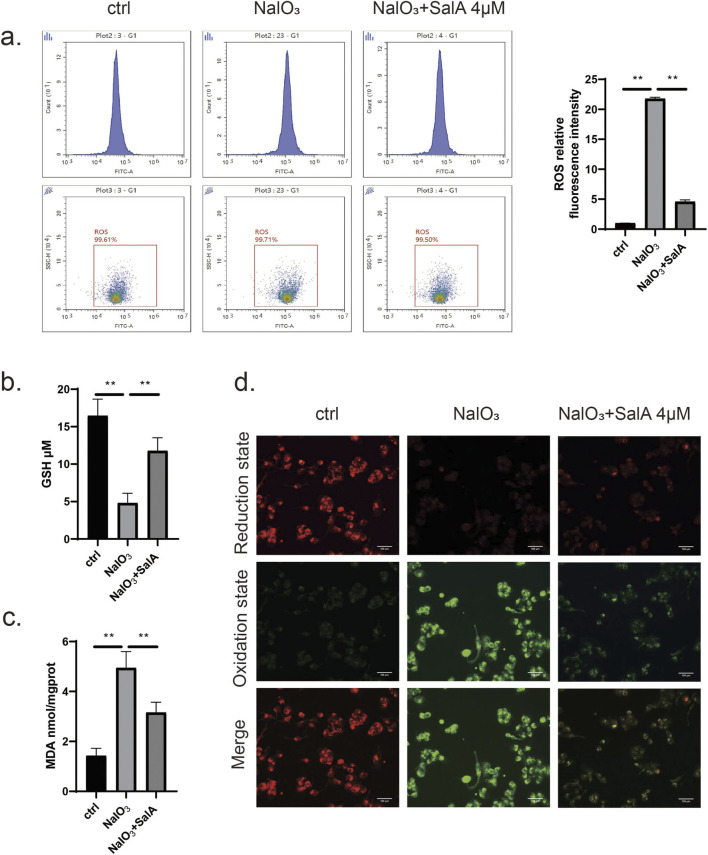
SalA Alleviates NaIO_3_-Induced Oxidative Stress and Lipid Peroxidation **(a)** Flow cytometric measurement of intracellular reactive oxygen species (ROS) using DCFH-DA. **(b)** Glutathione (GSH) levels quantified by a colorimetric assay. SalA reversed the NaIO_3_-induced depletion of GSH. **(c)** Content of malondialdehyde (MDA), a marker of lipid peroxidation. SalA markedly suppressed the MDA accumulation triggered by NaIO_3_. **(d)** *In situ* visualization of lipid peroxidation with the fluorescent probe C11-BODIPY^581^/^591^. The NaIO_3_-induced fluorescence shift from red (reduced) to green (oxidized) was effectively counteracted by SalA. Data present mean ± SD (n = 3 independent experiments). *p < 0.05, **p < 0.01.

As ferroptosis is driven by iron-dependent oxidative stress, we next assessed relevant markers. Flow cytometric analysis showed that NaIO_3_ exposure markedly increased intracellular ROS levels, an effect that was substantially mitigated by SalA pretreatment (Figure 3a). Furthermore, SalA treatment restored the NaIO_3_-induced depletion of glutathione (GSH), a key antioxidant (Figure 3b). Concurrently, treatment with SalA led to a significant reduction in levels of malondialdehyde (MDA), an indicator of lipid peroxidation (Figure 3c). Using the fluorescent probe C11-BODIPY^581^/^591^, we observed that NaIO_3_ induced a pronounced shift from red to green fluorescence, indicating elevated lipid peroxidation, which was suppressed by SalA (Figure 3d). These results demonstrate that SalA alleviates oxidative damage and inhibits lipid peroxidation in NaIO_3_-induced ferroptosis.

### 3.4 SalA suppresses ferroptosis via the SLC7A11/GPX4 axis in ARPE-19 cells

**FIGURE 4:**
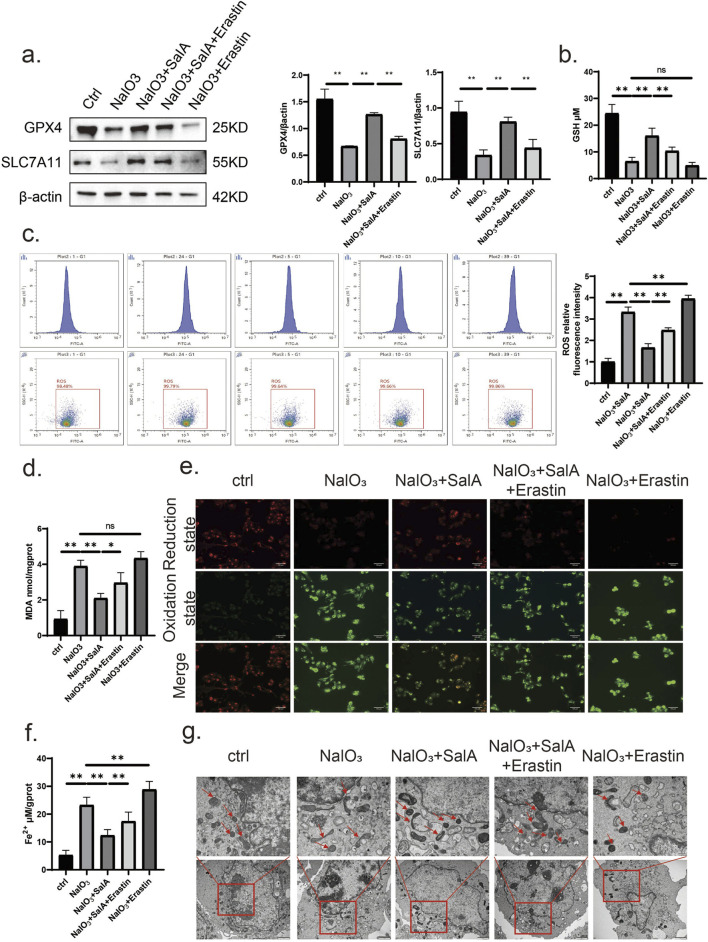
SalA suppresses ferroptosis via the SLC7A11/GPX4 Axis in ARPE-19 Cells. **(a)** Western blot analysis demonstrating that SalA upregulates GPX4 and SLC7A11 expression in NaIO_3_-exposed cells, an effect largely blocked by co-treatment with the SLC7A11 inhibitor erastin. **(b)** GSH levels. Erastin prevented the SalA-mediated restoration of GSH under NaIO_3_-induced stress. **(c–e)** Erastin significantly reversed the ameliorative effects of SalA on oxidative damage markers: **(c)** intracellular ROS levels, **(d)** MDA content, and **(e)** lipid peroxidation visualized by BODIPY^581^/^591^ fluorescence shift. **(f)** Intracellular Fe^2+^ quantification. Erastin exacerbated iron accumulation in NaIO_3_-treated cells and counteracted the iron-chelating activity of SalA. **(g)** Representative TEM micrographs showing mitochondrial ultrastructure. Red arrows indicate ferroptotic features, including mitochondrial shrinkage and increased membrane density. Erastin intensified NaIO_3_-induced mitochondrial damage and blocked the protective effect of SalA. Data present mean ± SD (n = 3 independent experiments). *p < 0.05, **p < 0.01.

To elucidate the mechanism by which SalA inhibits ferroptosis, we employed erastin, a selective inhibitor of SLC7A11. Western blot analysis revealed that SalA upregulated the protein expression of GPX4 and SLC7A11 in NaIO_3_-exposed cells, and this induction was abolished by erastin co-treatment (Figure 4a). Consistent with this, erastin blocked the SalA-mediated restoration of GSH levels (Figure 4b). Moreover, the protective effects of SalA against ROS accumulation, MDA content, and lipid peroxidation were all abrogated by erastin co-treatment (Figures 4c–e). Erastin also further enhanced intracellular Fe^2+^ accumulation (Figure 4f) and exacerbated NaIO_3_-induced mitochondrial damage (Figure 4g). Collectively, the fact that erastin alone exacerbated NaIO_3_-induced injury, while also abolishing the protective effects of SalA, corroborates the pivotal role of the SLC7A11/GPX4 axis in ferroptosis defense and confirms that SalA acts primarily through this pathway.

### 3.5 SalA attenuates NaIO_3_-induced ferroptosis in a mouse model of AMD

**FIGURE 5:**
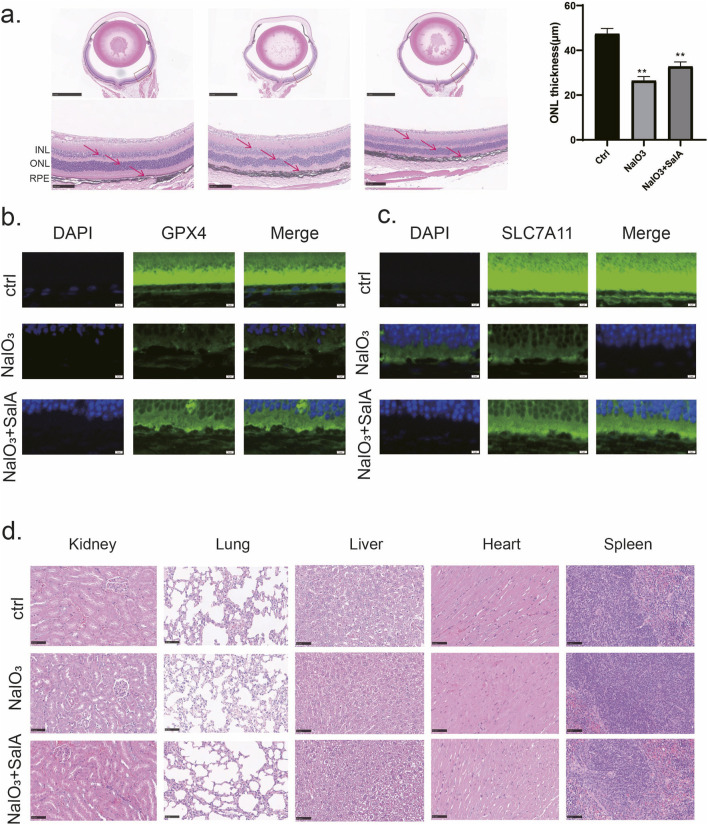
SalA attenuates NaIO_3_-induced ferroptosis in a mouse model of AMD **(a)** Representative hematoxylin and eosin (H&E)-stained retinal sections. NaIO_3_ administration induced RPE disruption and outer nuclear layer (ONL) thinning, which were markedly ameliorated by SalA treatment. **(b,c)** Immunofluorescence staining shows increased expression of **(b)** GPX4 and **(c)** SLC7A11 in the RPE and photoreceptor layers of SalA-treated mice compared to the NaIO_3_ group. **(d)** No significant toxicity or histopathological changes were observed in H&E-stained sections from the heart, liver, spleen, lung, and kidney across all groups. Data represent at least six biological replicates. Scale bars: 100 μm **(a)** 5 μm **(b,c)** 50 μm **(d)**. *p < 0.05, **p < 0.01.

We further evaluated the anti-ferroptotic efficacy of SalA in a NaIO_3_-induced mouse model of AMD. Histological analysis by H&E staining showed that NaIO_3_ administration induced significant retinal pigment epithelium (RPE) damage and outer nuclear layer (ONL) thinning. These pathological changes were substantially attenuated by SalA pretreatment (Figure 5a). Immunofluorescence (IF) analysis further indicated that SalA elevated the expression of SLC7A11 and GPX4 in retinal tissues (Figures 5b,c), consistent with activation of an anti-ferroptotic pathway. Safety evaluation via H&E staining of major organs (heart, liver, spleen, lung, and kidney) revealed no notable morphological abnormalities across all groups (Figure 5d), indicating good biocompatibility of SalA at the administered dose. These *in vivo* findings confirm that SalA protects against NaIO_3_-induced RPE ferroptosis and structural damage.

**FIGURE 6:**
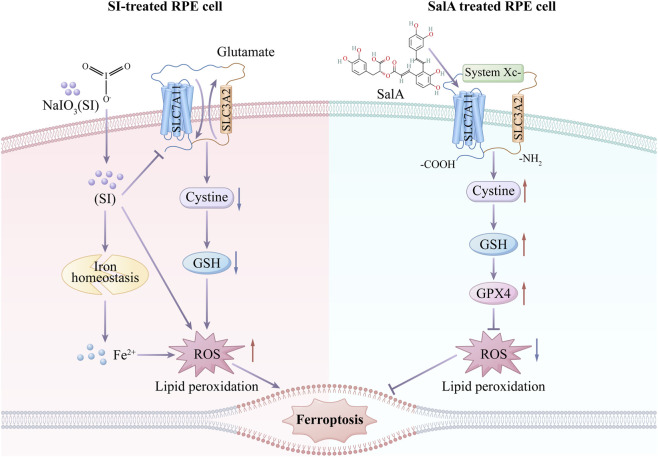
Proposed mechanism of SalA in inhibiting NaIO_3_-induced ferroptosis in RPE cells.

The left panel outlines the ferroptotic cascade initiated by NaIO_3_, marked by intracellular iron overload, GSH depletion, and ROS elevation, culminating in lethal lipid peroxidation. In contrast, the right panel demonstrates how SalA counteracts this process by activating the SLC7A11/GPX4 antioxidant axis. This activation promotes cystine uptake and GSH synthesis, thereby restoring GPX4 activity to scavenge ROS, suppress lipid peroxidation, and ultimately preserve RPE cell viability. This schematic integrates key molecular and cellular findings from the present study, as illustrated in Figure 6.

## 4 Discussion

Our study provides compelling evidence that SalA protects against NaIO_3_-induced RPE injury in both *in vitro* and *in vivo* settings. Mechanistically, we demonstrate that SalA attenuates ferroptosis—an iron-dependent form of regulated cell death—primarily by activating the SLC7A11/GPX4 antioxidant axis, thereby correcting iron dyshomeostasis and suppressing lethal lipid peroxidation.

Ferroptosis is driven by the failure of the GSH-GPX4 system and subsequent iron-catalyzed lipid peroxidation (Dixon et al., 2012; Jiang et al., 2021). Our results identify SalA as a potent modulator of this pathway, restoring the expression of key ferroptosis suppressors (SLC7A11, GPX4, FTH1) and inhibiting promoters (ACSL4, TFR2). Critically, the functional necessity of the SLC7A11/GPX4 axis was unequivocally established using its selective inhibitor, erastin, which blocked all protective effects of SalA. The observation that erastin co-treatment reversed SalA’s protection (Figures 4a–g), together with the efficacy of the NaIO_3_ + Erastin control group, robustly demonstrates that this axis is central to SalA’s action. Therefore, we conclude that SalA exerts its primary cytoprotective effect by activating the SLC7A11/GPX4 system. This places SLC7A11, the cystine/glutamate antiporter essential for GSH synthesis, as a critical molecular target of SalA. Our data suggest that by promoting cystine uptake, SalA ensures sufficient substrate supply for glutathione synthesis, ultimately enabling GPX4 to efficiently detoxify lipid hydroperoxides and maintain membrane integrity (Koppula et al., 2021; Ursini and Maiorino, 2020).

Our findings align with and extend the growing recognition of ferroptosis in AMD pathogenesis, which is linked to iron accumulation and lipid peroxide toxicity (Dixon et al., 2012; Kaarniranta et al., 2019; Wong et al., 2007). We build upon this foundation by identifying SalA, a natural phenolic compound derived from *Salvia miltiorrhiza*, as an effective ferroptosis inhibitor in the retinal context. While synthetic inhibitors are well-known (Friedmann Angeli et al., 2014), SalA represents a natural multi-target candidate, concurrently enhancing cellular antioxidant capacity (via SLC7A11/GPX4) and chelating labile iron, as suggested by reduced Fe^2+^ levels and upregulated FTH1. This dual-action mechanism is advantageous for addressing the multifactorial pathology of AMD. Elucidating the upstream mechanism by which SalA activates this axis is an important future direction; the potential involvement of the transcription factor Nrf2 is a promising candidate (Dodson et al., 2019; Ma, 2013).

The translational significance of our work is strongly supported by the *in vivo* data. SalA administration effectively preserved retinal structure, attenuated RPE damage, and upregulated SLC7A11 and GPX4 expression, corroborating our cellular findings. Importantly, no significant histopathological toxicity was observed in major organs, underscoring its favorable safety profile, consistent with the traditional use of *S. miltiorrhiza* (Jia et al., 2019; Tang et al., 2021; Zhao et al., 2023). Given the limited treatment options for dry AMD, our work establishes SalA as a promising lead compound. Future studies should focus on optimizing its ocular bioavailability (e.g., through novel drug delivery systems (Gaudana et al., 2010)) and evaluating its efficacy in chronic or multi-factorial AMD models (Pennesi et al., 2012).

We acknowledge several limitations. First, the acute NaIO_3_ model was used, testing SalA in other models (e.g., genetic or diet-induced) would enhance the generalizability of our conclusions. Second, a key functional correlate-electrophysiological data (e.g., ERG) linking structural rescue to visual function-is absent and should be a priority in future studies. Third, While the ARPE-19 cell line is widely used, it does not fully replicate the primary RPE physiology. Fourth, although erastin is a validated pharmacological tool, genetic approaches (e.g., SLC7A11 knockdown or knockout using siRNA or CRISPR-Cas9) (Hu et al., 2023) would provide more definitive evidence. Furthermore, while we focused on the SLC7A11/GPX4 axis, ferroptosis can be regulated through parallel pathways, such as the FSP1-CoQ10 system (Bersuker et al., 2019). Investigating a potential interaction between SalA and these alternative pathways would provide a more comprehensive mechanistic understanding.

## 5 Conclusion

In summary, this study provides compelling evidence that SalA attenuates NaIO_3_-induced RPE injury by inhibiting ferroptosis, primarily through activating the SLC7A11/GPX4 antioxidant pathway to alleviate iron overload and lipid peroxidation. These results elucidate a previously unknown cytoprotective mechanism of SalA and underscore the therapeutic relevance of ferroptosis inhibition in AMD. Consequently, SalA emerges as a highly promising natural product candidate, warranting further exploration both to deepen mechanistic understanding and to advance its translational development for retinal degenerative diseases.

## Data Availability

The original contributions presented in the study are publicly available. This data can be found here: https://www.ncbi.nlm.nih.gov/sra/PRJNA1467099, accession number PRJNA1467099.
